# A New Way of Analysing Dreams on Its Profoundest Level: The Development of Motif Analysis and Phase Model (MAP) as an Extension of Structural Dream Analysis (SDA)

**DOI:** 10.3390/bs14080658

**Published:** 2024-08-01

**Authors:** Patrick Jenni, Christian Roesler

**Affiliations:** 1 Psychological Institute, Zurich University of Applied Sciences, 8004 Zurich, Switzerland; 2 Department of Psychology, Catholic University Freiburg, 79104 Freiburg, Germany; christian.roesler@kh-freiburg.de

**Keywords:** structural dream analysis, motif analysis and phase model, psychotherapy, dream, process model, waking life, child motif, psychoanalysis

## Abstract

Dream research today assumes that there is a connection between dreams and waking life. However, the structural alteration of dream motifs in connection with the psychotherapeutic process and waking life has not yet been researched extensively. This study depicts the development of the new Motif Analysis and Phase Model (MAP), a dynamic method which allows research on the previous aspects. The following question was investigated as an accompanying key issue: can a connection be established between the course of the dream patterns and the agency of the dream ego as well as the dream contents and the course of the psychotherapies of the dreaming person as a whole? Four hypotheses were formulated and tested. The data material consists of 217 dreams of a male test subject. The motifs were analysed using Structural Dream Analysis (SDA) at first. Thereafter, the content was linked to the test subject’s waking life in a guided interview. The findings show a strong connection between the dream content and the psychotherapies as well as the test subject’s waking life. Five motifs with structural changes were found, through which the Phase Model with four phases was developed. At turning points, the transformative child motif also appears in the dreams. The course of the dream patterns and agency of the dream ego, however, has not changed. The results, the method and the generalisability were critically discussed and recommendations for future research were formulated.

## 1. Introduction

The assumption that dreams contain meanings that are closely related to waking life is the subject of profound research in recent dream research [1,2]. Likewise, working with dreams is helpful in psychotherapeutic treatment and can be used effectively in the psychotherapeutic process [3,4]. Modern dream theory goes back to Freud’s work The Interpretation of Dreams [5], in which he interpreted patients’ dreams and saw them as part of their treatment. This understanding still plays an important role in the psychoanalytic tradition within the treatment of mental illnesses [6,7].

Freud assumed that the treating psychotherapists were responsible for the final interpretation of the dream. The assumption that occurring symbols have different meanings from person to person and must therefore be interpreted subjectively by the dreaming person is reinforced by Carl Gustav Jung’s theoretical arguments described later. This aspect is also important for the present study, as no generally valid conclusions can be drawn at the level of symbols with their subjective content, whereas this is possible for the motifs and its structural appearance in dream sequences.

Jung saw the psyche as an autopoietic system that regulates itself. Dreams are spontaneously appearing images of the current situation in which the psychic life of the dreaming person is reflected. The meaning of these images is represented by symbols [8]. He assumed that the dream seeks to convey unlived parts of the personality, thereby providing the dreaming person with the opportunity to grow in their personal development [9].

For example, Jung describes the appearance of the child motif as a symbol of transformation that occurs “empirically in spontaneous and therapeutically triggered individuation processes” [10] (p. 194). Also, an “essential aspect of the child motif is its future character. therefore the appearance of the child motif usually signifies an anticipation of future developments” [10] (p. 178). According to Jung, the transformative child motif also anticipates transformations in the psychotherapeutic process and is described as an archetype of change [10]. Roesler [11] succeeded for the first time in providing empirical evidence for the existence of the child motif as a transformative indicator signalling a change in the psychotherapeutic process. As the child motif plays a central role in this study, it has been explained in detail here.

Jung also described theoretical considerations on the concept of the symbol, which for him represented a visual part of the psychic world. In contrast to a sign, such as a stop sign, this does not contain any explicit meaning but remains vague [9]. It thus offers the dreaming person the opportunity to attribute qualities to the symbol that arise from the unconscious. The concept of the symbol is not clearly distinguished from that of the motif in Jung’s theoretical arguments. However, this distinction is important for this study and is explained in the methods section.

In the 1970s, the first research was carried out to investigate the content and meaning of dreams. Research by Kramer et al. [12] showed that there is a connection between dreams and waking life and that dreams are representations of non-random events that reflect a person’s everyday life. Fisher and Greenberg [13,14] also found a connection between dreams and the waking life. Cartwright [15] was able to show that dream content changes during the period of psychotherapy. According to Ermann [16], dreams thus show psychological motifs as well as meaningfulness and contain psychotherapeutic value. These findings support the continuity hypothesis, which states that the content of dreams is related to the experiences and challenges of waking life [17,18,19,20].

Hall and Van de Castle [21] and Hall and Norby [22] were the first to carry out a systematic study of dream content. They developed a classification system based on the scientific criteria of objectivity, reproducibility, and generalisability. The development of the contingency analysis by Hall and Norby [22] based on this was able to show that it is possible to create a personality diagnosis purely based on dream content, which has a clear connection with the psychological living environment of the test subjects. Roesler [23], however, criticises this classification system for categorising very different content and for not being able to capture the meaning of a dream or dream series by counting the number of symbols and motifs that appear. Therefore, he developed the method of Structural Dream Analysis (SDA), which is described in detail in the methods section.

### Research Question and Hypotheses

Due to the experimental nature of this study, the research question is formulated in general terms to accommodate the fact that new fields of structural dream research are being explored. The question: is it possible to establish an overall connection between the dream motifs and the course of the dreamer’s psychotherapy? In addition to the research question, the following hypotheses have been defined: (I). One or more recurring motifs are found within the dream series, which structure changes over time. (II). The central motifs are connected to the waking life of the dreaming person. (III). The transformative child motif appears in the dreams at turning points. (IV). In the course of psychotherapy, the dream patterns and agency of the dream ego move from patterns 1, 2 and 3 to patterns 4, 5 and 6.

## 2. Materials and Methods

The methodological approach is based on SDA. As individual motifs have never been analysed to this extent within SDA, the development process that enabled the motifs to be analysed and ultimately led to the formulation of the new method is described later.

### 2.1. Structural Dream Analysis

The Structural Dream Analysis research method examines the meaning and structure of dream content that is dreamt during psychotherapeutic treatment. It was developed by Roesler [23,24,25,26,27] and uses a coding system that enables the structural changes in dreams to be analysed qualitatively and quantitatively regardless of the researcher or school of origin. Two types of coding are used: dream type and agency of the dream ego. The six codes of the dream type describe the structure of a dream series (e.g., performance demands on the dream ego, mobility dreams) while the agency of the dream ego describes the interaction of the dream ego with the dream content. Reliability tests by Gees [28], Kissling [29], Roesler [25], Widmer [30] and Zander [31] show that the coding has a moderate to strong interrater reliability of Cohen’s kappa values ranging *k* = 42–82.

Research on psychoanalytic therapy assumes that, if successful, it strengthens the ego strength of the person receiving treatment [32,33,34,35]. SDA works with the hypothesis that the increase or decrease in dream patterns and agency of the dream ego is accompanied by a change in the person’s ego strength. There is strong evidence that this assumption can be confirmed [24,25,27,28,29,30,31]. During the research on SDA, a special category has been defined that encodes transformative dreams whose content often includes, among other things, the appearance of a child who needs to be cared for by the dream ego [28,29,36].

As Roesler [11] was able to demonstrate empirically for the first time, it is possible that the child motif can accompany transformative processes within a dream series and thus in the course of therapy. The transformative child motif has also been previously researched by Stolp [37] and is also part of the investigation in this study. Research into the motif of the child using SDA has shown that it is possible to analyse motif structures in addition to the metaphysical analysis of a dream structure. As no manualised procedure has yet been defined, this study formulates a standardised dynamic procedure for investigating the motif structure of dream series.

### 2.2. Motif Analysis and Phase Model (MAP)

In order to be able to analyse motifs in a dream, the term motif was defined for the first time for this study. A motif within a dream consists exclusively of a noun (e.g., forest, river, house). Names (persons, places, etc.) are not included unless they are converted into nouns, e.g., father for Wolfgang or city for Zurich. The term motif must be distinguished from the term symbol, as the latter has a special meaning within analytical psychology and differs in terms of content: a motif can be grasped in concrete terms and can therefore be distinguished from other motifs, whereas a symbol remains vague [9].

There is also a hierarchy within the motifs. A motif can be summarised by combining motifs that have a similar meaning. For example, the motif of water can be analysed in a dream series. This summarises the motifs of brook, masses of water, fish, flood or swimming pool and is considered the main motif. If it now becomes apparent that in a dream series the motif of the fish, which is contained in the main motif of water, is mentioned in a more differentiated way in the dreams, e.g., with the motifs of fishing, fish trap, whale or trout, the motif of the fish becomes a new main motif and can be analysed structurally accordingly. The motif of water can be neglected, as the aim is to achieve the greatest possible depth of differentiation. This mechanism makes it possible to conduct the analysis dynamically and to apply it to any dream series and motif structure while being as close as possible to the dream content.

**Figure 1:**
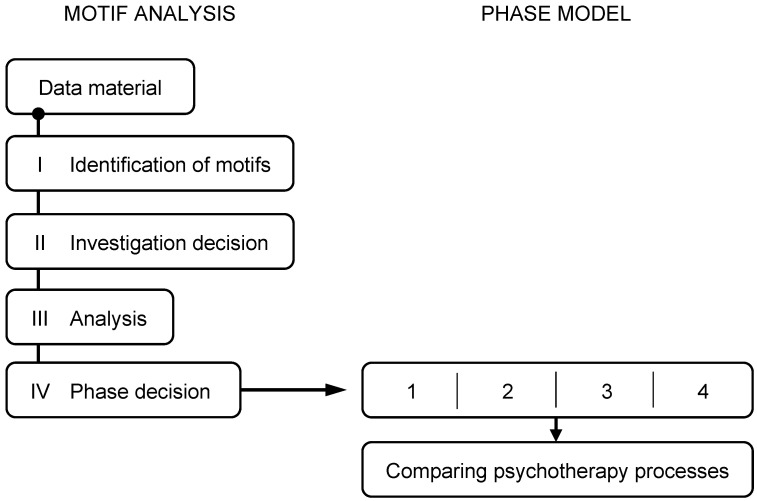
Visualisation of the analysis process conducted by the Motif Analysis and Phase Model (MAP). The process starts with data material. (1) prodromal phase, (2) aggregation phase, (3) confrontation phase, (4) integration phase.

In a first step, as described in Figure 1, all words that met the previously described criteria were counted and placed in a hierarchical order, starting with the most frequent occurrence. A total of 13 motifs descended from the data material used for this study were identified for further analysis.

The second step serves to economise the analysis. As 13 motifs would have meant too much effort for the analysis, the selection was limited: all motifs that occurred more than 30 times, all localities and all real-life persons were excluded. Motifs were also condensed, e.g., the motif of the car, which occurs 15 times and could not be further differentiated, was integrated into the main motif of locomotion. Eight motifs were identified for further analysis: mother, locomotion, water, house, father, child, girl and master. This step can be organised differently or omitted depending on the study design or data material.

The third step involves analysing the content of the motifs. For each dream containing one of the eight motifs, the affects of the dream ego, the general mood of the dream, recurring themes and recognisable patterns were noted. Table 1 provides an overview of this analysis based on the motif of the girl.

The fourth step divides the motifs into phases that can be distinguished from one another. Those motifs that could not be divided into phases were excluded from the analysis. Three motifs were excluded, so that the following were used: locomotion, water, father, girl and master. The latter exhibit a change in its four phases and are therefore considered to be motifs with structural change.

The phases of the motifs were then compared with each other over time. Also, the turning points within psychotherapy and the subject’s waking life were compared. Turning points are defined as conspicuous changes within the temporal course of a dream series or events that are labelled as such by the dreaming person. The turning points were surveyed in a semi-structured interview. In addition to the turning points, the motifs that showed a phase change were also asked about their significance for the test person.

### 2.3. Data Material

The material analysed includes 217 written dreams of a male test subject. The period of the records is between December 1969 and November 1976 and includes two psychotherapeutic treatments as part of the test subject’s training analysis, which he underwent during his training as a psychotherapist. The psychotherapy was conducted by two Jungian analysts; the first was male, and the second was female.

## 3. Results

As can be seen in the Methods section, five motifs emerged from the motif analysis, which came into question for a chronological progression of the phases. The third and fourth steps of the analysis are shown in Table 1.

### 3.1. Phase Model

The phases 1 to 4 shown in Table 1 occurred like this in the data material. This made it possible to formulate a new phase model. The phases were named as follows: phase 1, prodromal phase; phase 2, aggregation phase; phase 3, confrontation phase; phase 4, integration phase. The different phases are described below.

Phase 1: Based on the pathological concept of the prodrome, which describes a preliminary stage of an illness, the motif is recognisable as such in the prodromal phase and appears in the recording. However, it remains in its previous form. The dream ego shows a predominantly uniform interaction and is focused, with little deviation, on one aspect of the motif. The handling of the motif appears to be less varied, which is also reflected in the affects, the mood, the patterns and the themes (step 3 of the motif analysis) of the individual dreams. The interactions are predominantly defensive, which means that the dream ego reacts to the motif and its dynamics and shows little or no proactive behaviour.

Phase 2: In the aggregation phase, the phenomenology of the motif begins to change. The way the dream ego deals with the motif also changes. An affective and atmospheric enrichment (aggregation) and differentiation is visible. The dream ego recognises further aspects of a motif in this phase, whereas this enrichment is not recognisable in the prodromal phase. The dream ego also shows a greater spectrum of affectivity and can therefore deal with the motif in a more differentiated way. The differentiation and enrichment are stimulated by emerging persons or motifs in a way that the dream ego is able to recognise alternative ways of dealing with the motif in the sense of model learning and applying them in the next phase.

Phase 3: After the phase of aggregation, the phase of confrontation begins. This phase is characterised by an increasing interaction of the dream ego with the motif. In contrast to the previous phases, the dream ego recognises its own wishes and needs in this phase, stands up for them and pursues them in the interaction with the motif. The actions can be aggressive, for example, through a violent confrontation, but can also be peaceful. There is more interaction between the dream ego and the motif than is visible in the aggregation phase. In the phase of aggregation, the dream ego is able to apply the new insights about the motif learnt during the aggregation phase in its interaction with the motif.

Phase 4: In the integration phase, the motif is harmonised. Previous tensions and conflicts, and in some cases interactions, are now significantly lower in number and intensity. In this phase, the dream ego can react to the motif on its own initiative and according to its own wishes and ideas and lives in harmony with its own needs. There are balanced, less conflictual affects and the motif is often no longer a central component of the dream events. The difference to the prodromal phase is that, on the one hand, the phenomenology of the motif and the way the dream ego deals with the motif has changed over time. On the other hand, the motif is significantly less conflictual and the affects that emerge in the dreams are more balanced.

**Figure 2:**
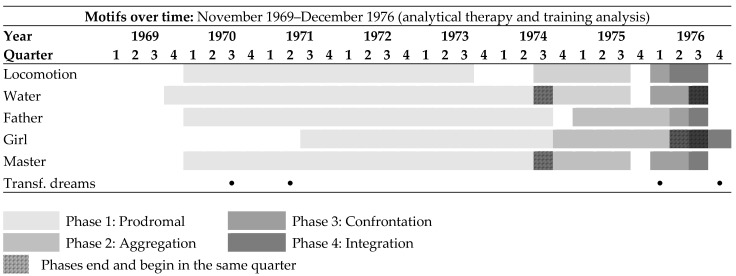
Only motifs with structural changes are listed. Motifs are sorted in descending order of frequency of occurrence. The dots indicate a transformative dream during this quarter.

Figure 2 shows that the four phases can be equated with each other distinctly over time and change at the same points in their phases. The occurrence of transformative dreams according to the coding of SDA was also integrated into Figure 2 in order to see whether these occur at phase transitions. This is the case for two such dreams. A phase transition was defined descriptively as a turning point. The guided interview showed that the test subject reported two turning points in his life that correspond to phase transitions.

On one hand, such a turning point occurred during the transition from the prodromal phase to the aggregation phase in 1973 and 1974, when the test subject changed the therapeutic treatment person and from then on experienced an appreciative and strengthening therapeutic relationship. The second turning point in the test subject’s life occurred at the end of the integration phase in 1976, when he completed treatment. The transformative child motif was also evident at both turning points.

### 3.2. Transformation of Dream Agency within Phase Model

As can be seen in Figure 2 and especially in Table 1, the dream ego’s room for action changes significantly over the course of the four phases. In the first two phases, passive activity of the dream ego predominates. The dream ego remains passive in the first phase (prodromal) and shows a one-sided approach to the motif. In the second phase (aggregation), the passivity continues, in that although further information about the motif is collected, the way it is dealt with does not yet change. The room for action increases in the third phase (confrontation) as the dream ego gains autonomy and initiative. In the fourth phase (integration), the active behaviour of the dream ego continues, and the motif increasingly fades into the background.

This dynamic is consistent with the results of SDA, which show that the ego strength of the dream ego in form of the agency of the dream ego increases in the course of psychotherapy if the treatment was able to bring about an improvement in the patient’s symptoms. While at the beginning of treatment the dream ego acts mainly passive and shows little initiative, at the end of treatment there is a strong increase in initiative. The interaction of the dream ego changes from passivity to activity [23,24]. This dynamic at the structural level of a dream series can be found in the MAP model at the level of motifs.

The increase in ego strength can be linked to the agency of the dream ego on the basis of the connection between the phases of MAP model. However, such a connection between ego strength and the dream pattern could not be confirmed statistically, as described in the discussion section.

### 3.3. Quantitative Calculation Based on SDA

The statistical calculation of the dream patterns using the Spearman rank correlation did not show a significant result (*p* = 0.50). The calculation of the agency of the dream ego was also not significant (*p* = 0.16). The effect sizes according to Cohen for the dream patterns (*r* = 0.046) and agency of the dream ego (*r* = 0.095) were statistically insignificant.

## 4. Discussion

The application of the MAP model to the dream series has identified five motifs that show a change over time: locomotion, water, father, girl and master. It turned out that the changes in the motifs over time are almost identical and can be divided into four phases. The new Phase Model was formulated based on this categorisation. In the guideline interview, the test subject was asked about the motifs with structural change and the course of his psychotherapeutic treatments.

It emerged that all five motifs were related to the test subject’s waking life. Three of them, the motif of the girl, father and master, also show a strong thematic connection between the structural change in the motifs and the test subject’s waking life. The test subject also confirmed that the therapeutic relationship changed during the period under investigation, which was reflected in his waking life as well as in the dream series. The content of the psychotherapeutic process shows a strong connection with the dream series and the test subject’s waking life, which can also be seen in central turning points (Figure 1).

It is currently not possible to say with certainty whether the dream content has influenced experiences in the subject’s waking life, or vice versa. It is currently only possible to determine that the dream content and corresponding experiences in waking life occur in the same period of time. Further research is needed to confirm such a causality.

Based on the above findings, the question can be answered to the effect that overall, there is a strong connection between dream content and the course of psychotherapy. Hypothesis I can be confirmed: the results have shown that a structural change occurs over time for five of the eight most frequent motifs. This change can be divided into four phases, from which the new Phase Model has emerged. Hypothesis II can also be confirmed: the study shows that the five motifs that exhibit a structural change are related to the test subject’s waking life. The motifs of the girl, father and master show congruent correspondences between the structural change and the test subject’s waking life. Hypothesis III can be partially confirmed: four turning points can be identified. The transformative child motif appears at two of these four turning points: during the transition between the phase of aggregation and confrontation and at the end of the dream series. Hypothesis IV cannot be confirmed: the results show no significant change in the dream patterns and agency of the dream ego and the effect sizes are also not significant.

It has been shown that the connection between dream motifs and waking life is manifest, i.e., that the theme, for example that of authority in the motif of the master, can be found almost congruently in the test subject’s waking life. These results confirm Jung’s view that dreams have a direct connection to waking life and that they provide the dreaming person with impulses to promote the mastery of a certain topic in the waking life and to initiate personality development [9].

### 4.1. Therapeutic Relationship

It is evident that the change in the therapeutic relationship goes hand in hand with the change in the structure of the motif. The test subject was able to experience an appreciative relationship dynamic with the second treatment person, which is considered a central element of effectiveness in psychotherapy research [38]. The results indicate that inner processes were also triggered at the time of the change in treatment, which is reflected in the structural change in motifs. The test subject also reported in the guideline interview that a one-sided experience of his understanding of his identity as a man and father could be treated with the second treatment person.

The change in this understanding is expressed in the motif of the father, whose other characteristics could also be recognised more comprehensively by the dream ego during the aggregation phase. There is possibly a connection between the confrontation of his masculinity and the motifs of the master and father. In both motifs, there are increasing confrontations of the dream ego with the motifs that can be described as aggressive. The aspect of confrontation is possibly accompanied by a strengthening of the test subject’s masculinity, which empowers him to enter into confrontation in order to ultimately find a way of coping with the motif and the issues associated with it.

### 4.2. Transformative Child Motif

In the present study, the transformative child motif appears at important turning points and can further strengthen the theoretical assumption that it is possibly an archetype of change [10,11]. However, it must be noted that the motif appears at two other points in time that cannot be linked to a turning point. Further research into the transformative child motif is therefore necessary in order to fully confirm its habitual existence.

### 4.3. Analysis with MAP

The phases found result in the new Phase Model. It is possible to establish a connection with the results of Roesler [23], who was able to show a change in the structure of dreams from the second half of psychotherapeutic treatment. He describes that the dream structure in people with successful psychotherapeutic treatment shows a dominant repetitive pattern in the first half, which is linked to the dreamer’s psychological themes.

A similar pattern is recognisable in the prodromal phase: psychological subjects and challenges are depicted in the dreams and the repetitive approach of the dream ego is also dominant in this sense. In Roesler’s data, the transformative child motif appears in the half of the dream series, whereupon the second half is characterised by a change in pattern [11,23]. In Phase Model, this change can be recognised in two thirds of the dream series and is not announced or accompanied by the transformative child motif. However, it occurs at later turning points.

### 4.4. Ego Strengh According to SDA

It has been shown that the course of the test subject’s psychotherapy does not go hand in hand with the SDA hypothesis that the values change significantly from dream patterns 1, 2 and 3 to 4, 5 and 6 after successful psychotherapy and that there is thus an increase in ego strength. This may be related to the fact that the test subject did not seek psychotherapy because of psychological complaints, but for the purposes of his training as a psychotherapist and therefore the work on symptoms and ego strength was not central. It is also possible that the test subject already has a stable ego strength and that this has therefore not changed significantly as a result of the treatment.

The MAP model, on the other hand, can show that there is an increase in the active behaviour of the dream ego with motifs during the course of the therapy. This can hypothetically be interpreted as an increase in ego strength. It should also be noted that there is a statistical effect for the dream patterns as well as for the agency of the dream ego using SDA. A larger sample of dream series is necessary in future research in order to be able to prove the significance of this correlation.

### 4.5. Limitations and Outlook

As the motif analysis was defined and applied for the first time for this study, it is subject to some limitations. In general, it can be stated that the generalisability is not given due to the sample as an individual case and that studies on larger samples are indicated. This means that the statistical quality criteria for MAP model can be increased and examined quantitatively. Step three of the motif analysis offers a lot of interpretative leeway based on the subjectivity of the researcher. For future research, it may be indicated that the four categories be standardised, for example, by defining which affects can be distinguished from each other or when and how a pattern or recurring theme can be defined and distinguished from each other.

Step four is also the topic with subjectivity as to when the dreams in a series can be categorised into which phases of Phase Model. Further investigations into the quality factors are indicated for both steps. The categorisation of dreams into the four phases of Phase Model also offers a great deal of scope for interpretation at this stage, which can be designed differently depending on the person conducting the research. Reliability tests are also indicated for future research.

Research into the MAP model and the transformative child motif offers potential for investigating the question of whether such recurring motifs are archetypes, as described by Jung, and indicate changes within the psyche and in waking life. On the other hand, it can be of practical benefit if the connection between dream motifs and the psychotherapeutic process can be established in greater depth. This knowledge can serve as a diagnostic tool within a psychotherapeutic treatment and validate it as such. In conclusion, the present study shows promising results that opens new fields of structural dream research due to its experimental character.

## Figures and Tables

**Table 1:** Example of the change in structure in the motif of the girl.

	Phase 1	Phase 2	Phase 3	Phase 4
Dream-No.	57, 85, 90, 94	107, 109, 120, 134, 152, 156	157, 167, 182	191, 195, 210, 217
Affects	Fear, searching, timid	Excited, inhibited, astonished	Courage, criticising, rejecting	Interested, admiring, joy
Mood	Upset, powerful, pursuing	Sublime, dynamic, fast-paced	Promising, confrontational	Caring, fun
Themes	Shame	Devaluation/revaluation	-	-
Pattern	Dream ego does not dare to make contact with girls	Dream ego is more confident, interaction becomes more differentiated	Dream ego finds courage for sexual interaction, contact succeeds	The dream ego does not care if there are no sexual interactions

Note. The term “girl” refers to young women.

## Data Availability

The data that support the findings of this study are available from the corresponding author upon reasonable request.

## References

[1] Adam K.-U. (2006). Therapeutisches Arbeiten mit Träumen.

[2] Hill C.E. (1996). Working with Dreams in Psychotherapy.

[3] Ellis L. (2020). A Clinician’s Guide to Dream Therapy: Implementing Simple and Effective Dreamwork.

[4] DeCicco T.L., Donati D., Pini M. (2012). Examining dream content and meaning of dreams with English and Italian versions of the storytelling method of dream interpretation. Int. J. Dream Res..

[5] Freud S. (1900). The Interpretation of Dreams.

[6] Fonagy P., Kächele H., Leuzinger-Bohleber M., Taylor D. (2012). The Significance of Dreams: Briding Clinical and Extraclinical Research in Psychoanalysis.

[7] Fosshage J.L. (1997). The organizing functions of dream mentation. Contemp. Psychoanal..

[8] Jung C.G. (1971). Allgemeine Gesichtspunkte zur Psychologie des Traumes.

[9] Jung C.G. (1997). Traum und Traumdeutung.

[10] Jung C.G., Jung-Merker L., Rüf E. (2011). Die Archetypen und das kollektive Unbewusste. Gesammelte Werke.

[11] Roesler C. (2024). The Dream Motif of the Child as a Marker for Therapeutic Transformation.

[12] Kramer M., Hlasny R., Jacobs G., Roth T. (1976). Do dreams have meaning? An empirical inquiry. Am. J. Psychiatr..

[13] Fisher S., Greenberg R. (1977). The Scientific Credibility of Freud’s Theories and Therapy.

[14] Fisher S., Greenberg R. (1996). Freud Scientifically Reappraised: Testing the Theories and Therapy.

[15] Cartwright R.D. (1977). Night Life: Explorations in Dreaming.

[16] Ermann M. (2005). Träume und Träumen.

[17] Domhoff R.D. (2017). The invasion of the concept snatchers: The origins, distortions, and future of the continuity hypothesis. Dreaming.

[18] Levin R. (1990). Psychoanalytic theories on the function of dreaming. A review of the empirical dream research. Empir. Stud. Psychoanal. Theor..

[19] Pesant N., Zadra A. (2004). Working with dreams in therapy. What do we know and what should we do?. Clin. Psych. Rev..

[20] Schredl M., Kramer M., Gluecksman M. (2015). The continuity between waking and dreaming. Dream Research: Contributions to Clinical Practice.

[21] Hall C.S., Van de Castle R.L. (1966). The Content Analysis of Dreams.

[22] Hall C.S., Norby V.J. (1972). The Individual and His Dreams.

[23] Roesler C. (2019). Dream content corresponds with dreamer’s psychological problems and personality structure and with improvement in psychotherapy: A typology of dream patterns in dream series of patients in analytical psychotherapy. Dreaming.

[24] Roesler C. (2018). Structural dream analysis: A narrative research method for investigating the meaning of dream series in analytical psychotherapies. Int. J. Dream Res..

[25] Roesler C. (2020). Jungian theory of dreaming and contemporary dream research–findings from the research project ‘Structural Dream Analysis’. J. Anal. Psychol..

[26] Roesler C. (2021). Differences in dream content and structure between Japanese and Western dreams. Int. J. Dream Res..

[27] Roesler C., Lucius-Hoene G., Holmberg C., Meyer T. (2021). Structural dream analysis: A narrative methodology for investigating the meaning of dream series and their development in the course of psychotherapy. Illness Narratives in Practice: Potentials and Challenges of Using Narratives in Health-related Contexts.

[28] Gees A. (2021). Zusammenhang der Ich-Stärke im Traum und dem Psychotherapeutischen Prozess: Eine Quantitative und Qualitative Analyse mit der Methodik der Strukturalen Traumanalyse. Master’s Thesis.

[29] Kissling L. (2021). Untersuchung der Traummuster von Patienten mit Einer Gering Integrierten Persönlichkeitsstruktur—Eine Qualitative und Quantitative Analyse der Methodik der Strukturalen Traumanalyse. Master’s Thesis.

[30] Widmer D.B. (2019). Structural Dream Analysis: The Case of Amalie, X. Master’s Thesis.

[31] Zander P. (2020). Zusammenhänge von Traumstruktur und Therapiefortschritt—Strukturale Traumanalyse eines Historischen Einzelfalls. Master’s Thesis.

[32] Ermann M., Feidel R., Waldvogel B. (2001). Behandlungserfolge in der Psychotherapie.

[33] Poscheschnik G. (2009). Empirische Forschung in der Psychoanalyse—Vorbehalte und Vorteile. Psyche.

[34] Leichsenring F. (2002). Zur Wirksamkeit psychodynamischer Therapie. Ein Überblick unter Berücksichtigung der Kriterien der Evidence-based Medicine. Z. Psychosom. Med. Psychother..

[35] Leichsenring F., Poscheschnik G. (2005). Wirkungsnachweise psychoanalytischer und tiefenpsychologisch fundierter Therapie. Empirische Forschung in der Psychoanalyse.

[36] Radicevic L. (2021). Die Traumstruktur als Bühne für die Gesamtpersönlichkeit. Von, C.G. Jung und seiner Traumarbeit zu der Strukturalen Traumanalyse STA. Master’s Thesis.

[37] Stolp F. (2023). Veränderungsprozesse von Trauminhalten im Verlauf einer Psychotherapie. Master’s Thesis.

[38] Horvath A.O., Del Re A.C., Flückiger C., Symonds D. (2011). Alliance in individual psychotherapy. Psychotherapy.

